# Mitochondrial genome of *Trichagalma acutissimae* (Hymenoptera: Cynipoidea: Cynipidae) and phylogenetic analysis

**DOI:** 10.1080/23802359.2020.1721366

**Published:** 2020-02-07

**Authors:** Shuang Xue, Yuanchen Zhang, Shanshan Gao, Shaohui Lu, Jingshun Wang, Kunpeng Zhang

**Affiliations:** a College of Biology and Food Engineering, Anyang Institute of Technology, Anyang, Henan, China;; b College of Plant Protection, Henan Agricultural University, Zhengzhou, Henan, China;; c Henan Academy of Forestry, Zhengzhou, Henan, China

**Keywords:** Cynipidae, mitochondrial genome, *Trichagalma acutissimae*, phylogenetic analysis

## Abstract

*Trichagalma acutissimae* (Monzen) (Hymenoptera: Cynipidae) is a major pest of *Quercus variabilis* Blume in the Taihang Mountains in China. In this study, we sequenced and analyzed the mitochondrial genome (mitogenome) of *T. acutissimae*. This mitogenome was 16,078 bp long and encoded 13 protein-coding genes (PCGs), 22 transfer RNA genes (tRNAs), and 2 ribosomal RNA unit genes (rRNAs). The whole mitogenome exhibited heavy AT nucleotide bias (86.2%). Except for *nad4L* that started with TTG, all other PCGs started with the standard ATN codon. All 13 PCGs terminate with the stop codon TAA. Phylogenetic analysis showed that *T. acutissimae* got together with *Synergus* sp. with high support value, indicating the close relationship of these two genus. All five Cynipoidea species constituted a major clade and formed a sister group to Proctotrupoidea and Chalcidoidea.

Cynipoidea is the third-largest superfamily of parasitic Hymenoptera, which includes species exhibiting a wide range of life modes (Ronquist 1999). Most of the phytophagous Cynipidae can induce a great variety of galls, among them, are some of the most complex of all insect galls (Nieves-Aldrey et al. 2005). The Cynipini are restricted to plants of the family Fagaceae, predominantly oaks (*Quercus* spp.), on which they induce galls of diverse structures in leaves, buds, stems, flowers, fruits, and roots (Stone et al. 2002). *Trichagalma acutissimae* (Hymenoptera: Cynipidae) is one of the important pests harming afforestation plants *Quercus variabilis* and is very difficult to control by chemical pesticides.

Specimens of *T. acutissimae* were collected from Linzhou City, Henan Province, China (36°07′N, 113°43′E, October 2019) and were stored in Entomological Museum of Anyang Institute of Technology (Accession number AIT-E-TRI07). After morphological identification, total genomic DNA was extracted from tissues using DNeasy DNA Extraction kit (Qiagen, Hilden, Germany). The mitogenome sequence of *T. acutissimae* was generated using Illumina HiSeq 2500 Sequencing System (Illumina, San Diego, CA). In total, 6.2 G raw reads were obtained, quality-trimmed, and assembled using MITObim v 1.7 (Hahn et al. 2013). By comparison with the homologous sequences of other Cynipoidea species from GenBank, the mitogenome of *T. acutissimae* was annotated using the software Geneious R8 (Biomatters Ltd., Auckland, New Zealand).

The nearly complete mitogenome of *T. acutissimae* is 16,078 bp (Genbank accession, MN928529) in length and contains 13 protein-coding genes (PCGs), 22 tRNA genes, and 2 rRNA genes. The overall base composition of the mitogenome was estimated to be A 42.9%, T 43.3%, C 7.6%, and G 6.2%, with a high AT content of 86.2%. Compared with the ancestral insect mitochondrial genome, the mitogenome of *T. acutissimae* exhibits dramatic mitochondrial gene rearrangement, which is usually found in Cynipoidea species (Mao et al. 2015; Tang et al. 2019). Most PCGs of *T. acutissimae* had the conventional start codons ATN (five ATG, five ATT, and two ATA), with the exception of *nad4L* (TTG). All 13 PCGs terminate with the stop codon TAA. The lengths of *rrnL* and *rrnS* in *T. acutissimae* were 1396 and 853 bp, with the AT contents of 89.5 and 91.1%, respectively. The 22 tRNA genes vary from 64 bp (*trnT*) to 75 bp (*trnC* and *trnK*).

The phylogenetic tree was constructed using the maximum-likelihood method through raxmlGUI 1.5 (Silvestro and Michalak 2012) based on 13 mitochondrial protein-coding genes sequences. Results showed that the newly sequenced species *T. acutissimae* got together with *Synergus* sp. with high support value, indicating the close relationship between these two genera (Figure 1). All five Cynipoidea species constituted a major clade and formed a sister group to Proctotrupoidea and Chalcidoidea. In conclusion, the mitogenome of *T. acutissimae* is sequenced in this study and can provide essential DNA molecular data for further phylogenetic and evolutionary analysis of Cynipoidea.

**Figure 1.**
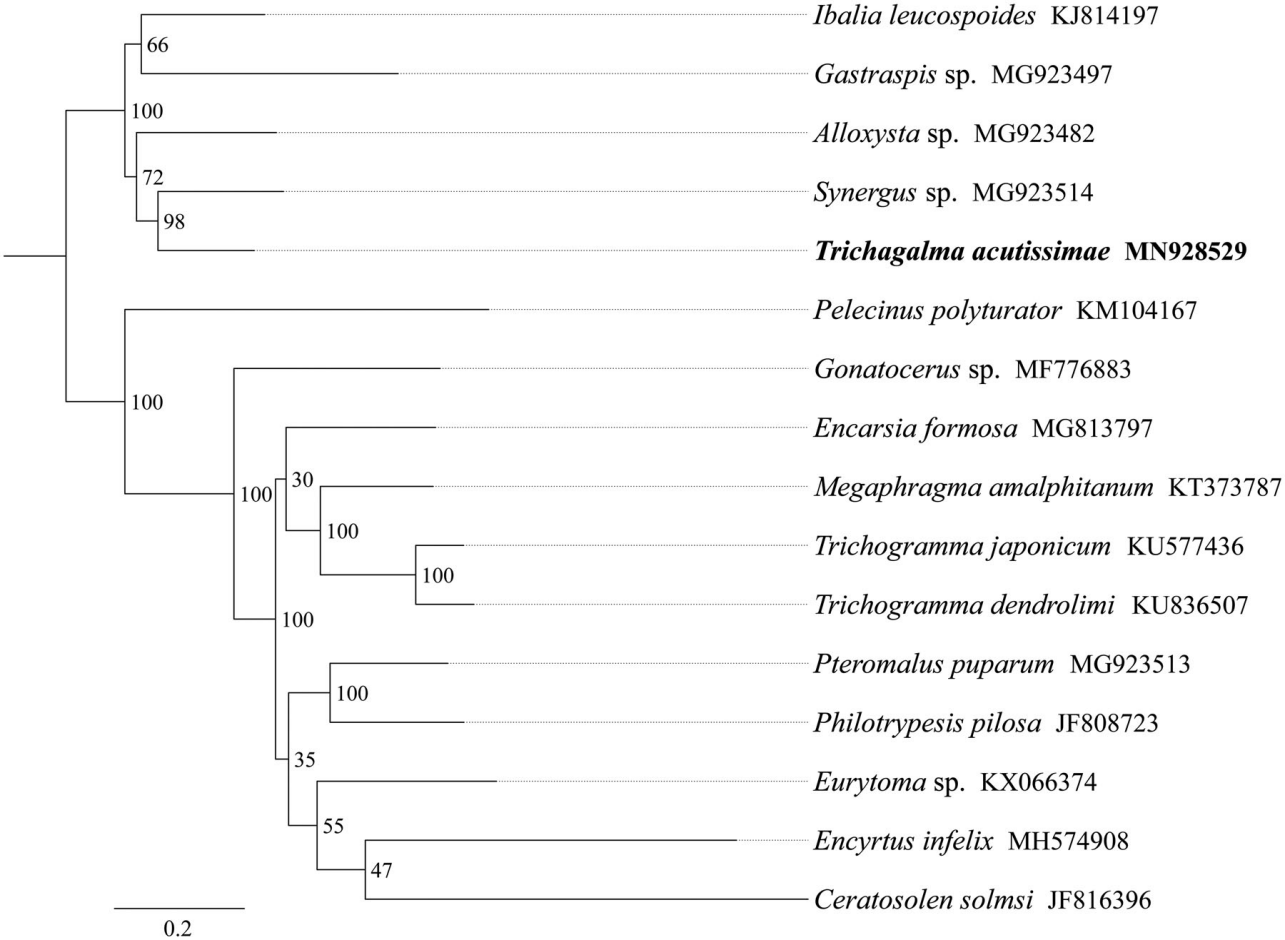
Phylogenetic relationships based on the 13 mitochondrial protein-coding genes sequences inferred from RaxML. Numbers on branches are Bootstrap support values (BS).
